# Haploid to diploid alignment for variation calling assessment

**DOI:** 10.1186/1471-2105-14-S15-S13

**Published:** 2013-10-15

**Authors:** Veli Mäkinen, Jani Rahkola

**Affiliations:** 1 Helsinki Institute for Information Technology, Department of Computer Science, University of Helsinki, Helsinki, 00014, Finland

## Abstract

**Motivation:**

Variation calling is the process of detecting differences between donor and consensus DNA via high-throughput sequencing read mapping. When evaluating the performance of different variation calling methods, a typical scenario is to simulate artificial (diploid) genomes and sample reads from those. After variation calling, one can then compute precision and recall statistics. This works reliably on SNPs but on larger indels there is the problem of invariance: a predicted deletion/insertion can differ slightly from the true one, yet both make the same change to the genome. Also exactly correct predictions are rare, especially on larger insertions, so one should consider some notion of approximate predictions for fair comparison.

**Results:**

We propose a full genome alignment-based strategy that allows for fair comparison of variation calling predictions: First, we apply the predicted variations to the consensus genome to create as many haploid genomes as are necessary to explain the variations. Second, we align the haploid genomes to the (aligned) artificial diploid genomes allowing arbitrary recombinations. The resulting haploid to diploid alignments tells how much the predictions differ from the true ones, solving the invariance issues in direct variation comparison. In an effort to make the approach scalable to real genomes, we develop a simple variant of the classical edit distance dynamic programming algorithm and apply the diagonal doubling technique to optimise the computation. We experiment with the approach on simulated predictions and also on real prediction data from a variation calling challenge.

## Background

In the study of human genetics, *variation calling *from high-throughput sequencing reads [1] is a revolutionary technique. Conceptually, the process is remarkably simple. Sequence random short fragments from donor DNA and align them to the reference (consensus) genome. Outside repetitive regions a good alignment is unique, hence if the resulting multiple alignment (read pileup) has columns where reads vote for something differing from the reference genome, the donor is very likely to actually contain this variant in his/her genome. Numerous methods have been proposed to fine-tune this simple scheme. One could argue that this problem is actually an enormous local multiple alignment problem, and so it is not surprising that methods trying to locally improve the alignment are able to improve the accuracy. This standard approach only captures small scale variations, and the methods for discovering large insertions, deletions, translocations, reversals, etc., are more involved [2].

Surprisingly there has been no systematic approach for studying the accuracy issue of variation calling predictions. With real data sets one can always resort to Sanger sequencing to validate the findings, but this is expensive. With simulated data sets one can compare to the ground truth and compute precision/recall statistics. A typical simulated data set is produced by first applying random mutations to a reference genome or selecting a random subset of a predefined set of known frequent variations in the population (or a mixture of these), to create an artificial donor genome. We consider here the setting of a diploid genome, where this simulation is conducted twice, taking into account heterozygousity/homozygousity information on the variants to create a realistic diploid genome. What we assume is that the alignment of the diploid genome to the reference is preserved. Then one can simulate a random pool of short reads from the artificial diploid genome with high enough coverage such that variation calling is plausible. Once a list of predicted variants is produced, one can match it to the list of variants used for creating the artificial diploid.

However, this direct comparison has the shortcoming that invariances among predictions are not properly considered. To see this, consider reference ACGGAAGGT, donor ACGGT, and let the simulated real variant be deletion of GAAG at position 3 (starting indexing from 0). Predicted deletion AAGG at position 4 results in the same donor, but pair-wise comparison of predicted and real variants would not reveal this. While these kind of simple invariances are easy to take into account, things get much more involved with split/merged predictions of various types. Clearly some kind of dynamic programming approach would be required to find best editing of the predicted variants to make them match the real ones. Here we assume that the allowed variant types are only insertions, deletions and substitutions, since rearrangements can always be modelled as series of the former type of operations.

Instead of correcting for the invariances in the manner sketched above, we propose to resort to a natural variant of the familiar sequence level full-genome alignment. The approach is as follows. First apply the predicted variations to the consensus genome to create as many haploid genomes that are necessary to explain the variations. Heterozygous variations are only applied on one haploid and homozygous variations on all. Second, align the haploid genomes to the (aligned) artificial diploid genomes allowing arbitrary recombinations. The intuition is that with short read mapping one cannot usually obtain much haplotype level information, but only determine whether the variations are heterozygous or homozygous. The haploid genome created by applying all non-overlapping predicted variants is better the closer it is to an arbitrary recombination of the diploid alleles. This is what the approach measures by edit distance. If there are overlapping predicted heterozygous variants, multiple haploid genomes are created and compared with a (different) recombination of the diploid alleles. Hence, the resulting haploid to diploid alignments tell how much the predictions differ overall from the true ones, solving the invariance issues in direct variation comparison. To make the approach scalable to real genomes, we develop a simple variant of the classical edit distance dynamic programming algorithm and apply the diagonal doubling technique to optimise the computation. We experiment with the approach on simulated predictions and on real prediction data from a variation calling challenge [3].

## Methods

### Problem

Let *B*^1 ^and *B*^2 ^represent diploid genome sequences generated by applying mutations to a reference sequence *P*. Let tables *m*^1 ^and *m*^2 ^store for each position in *B*^1 ^and *B*^2 ^the corresponding position in *P *defined by the multiple alignment of *P, B*^1^, and *B*^2^, which in turn is defined by how *P *is mutated to *B*^1 ^and *B*^2^. For example, the multiple alignment

   0123456 7 8

P  AGCTGAT-A-C

B1 ACCTGATCACG

B2 ACCTGCT-ACC

is defined by homozygous substitution G->C at 1st position of *P *, heterozygous substitution A->C at 5th position of *P *, heterozygous insertion of C after 6th position of *P *, homozygous insertion of C after 7th position of *P*, and heterozygous substitution C->G at 8th position of *P*. The corresponding mappings are

m1 01234566778

m2 0123456778

After simulating reads from *B*^1 ^and *B*^2^, let some variation calling method end up with the above list of variations. Then one can construct a haploid genome *A *by applying greedily all predicted mutations to *P *from left to right. In the general case, one could be left with overlapping mutations. Then one should construct another haploid genome applying all homozygous mutations as well; it is possible that there are more overlaps because of prediction errors [4], but since our simulated ground-truth is diploid, generating more predicted genomes is not beneficial in this case.

In our running example, all mutations can be applied simultaneously, as there are no overlapping heterozygous mutations. The result is *A *= ACCTGCTCACC, which can be aligned to the above multiple alignment with no errors by allowing the alignment to recombine *B*^1 ^and *B*^2 ^at the positions they share in common with *P*. We call such a recombination *a valid reference guided recombination*. The result is shown below (lower case showing the alignment of *A*).

A  ACCTGCTCACC

B1 ACCTGATcaCG

B2 acctgct-Acc

#### Invariance

To highlight the issue of invariance, let us consider a different reference *P *= GATCAATGAG, a single diploid *B *and a haploid *A *given by some variation calling method. Let us assume that *B *and *A *differ from *P *by the following variations

$$\begin{array}{ccc} \text{position} & \text{P} & \text{B}\\ 0 & \text{GA} & \text{G}\\ 3 & \text{C} & \text{CC}\\ 4 & \text{A} & \text{C}\\ 7 & \text{G} & \text{A} \end{array}\;\;\;\begin{array}{ccc} \text{position} & \text{P} & \text{A}\\ 1 & \text{A} & \text{T}\\ 2 & \text{T} & \text{C}\\ 3 & \text{C} & \text{CC}\\ 3 & \text{CA} & \text{C}\\ 7 & \text{G} & \text{A} \end{array}$$

The evaluation method used in Variathon 2013 [3] finds only two common variations: the one in position 3 and the one in position 7. Even if one was to use a method to match two invariant variants that make the same effect to the reference, this would not help in this example; only when the variants are applied altogether, the result is the same genome GTCCCATAAG:

P  GATC-AATGAG          P  GATC-AATGAG

B  G-TCCCATAAG          A  GTCCC-ATAAG

To allow approximate predictions and account for invariance, we propose to compute the unit cost (Levenshtein) edit distance between *A *and any recombination *B*^1 ^and *B*^2 ^according to their alignment.

The computation of such edit distance is an easy extension of the standard dynamic programming, and has been studied earlier under name *jumping alignments *in the context of amino acid sequences [5]. However, global alignment of full genomes is infeasible using standard quadratic time dynamic programming. Therefore we extend the diagonal doubling optimisation [6] to compute the edit distance in *O *(*dn*) time, where *d *is the least edit distance between the haploid and any valid reference guided recombination of the diploid genomes, and *n *is the maximum of the input sequence lengths.

### Algorithm

We simultaneously fill two dynamic programming matrices $\left(d_{i,j_{1}}^{1}\right)$ and $\left(d_{i,j_{2}}^{2}\right)$ such that $\left(d_{i,j_{1}}^{1}\right)$ (resp. $\left(d_{i,j_{2}}^{2}\right)$) gives the minimum edit distance between *A*[0 *... i*] and *R*[0 *... j'*] such that *R*[0 *... j'*] is a valid reference guided recombination of *B*^1^[0 *... j*_1_] and *B*^2^[0 *... j*_2_] ending at *B*^1 ^(resp. ending at *B*^2^). Filling the matrices is an easy extension of standard dynamic programming for edit distance: take minimum across the matrices at columns *j*_1 _and *j*_2 _such that *B*^1 ^and *B*^2 ^can recombine. Such *j*_1 _and *j*_2 _values have the property that

$$\begin{array}{c} m^{1}\left[j_{1}-1\right]=m^{2}\left[j_{2}-1\right]=j\;\text{or}\\ m^{1}\left[j_{1}-1\right]=m^{2}\left[j_{2}\right]-1=j\; \end{array}$$

and

$$m^{1}\left[j_{1}-2\right]\neq j\neq m^{2}\left[j_{2}-2\right]$$

This results into algorithm 1, where call *d *= diploid_align(*A, B*1*, B*2*, m*^1^*, m*^2^*, reference length*) returns the minimum edit distance *d *between *A *and any valid reference guided recombination of *B*^1 ^and *B*^2^.

Proof of correctness for Algorithm 1

**Definition 1**. Let *A *and *B *be strings. Now ed(*A, B*) is the Levenshtein edit distance between the strings *A *and *B*.

**Definition 2**. Let *A, B*^1 ^and *B*^2 ^be strings. *R_j _*is the reference guided recombination of *B*^1^[0 *... k*] and *B*^2^[0 *... v*] that minimises ed(*A, R_j_*), where *k *= max{*i | m*^1^[*i*] *≤ j*} (likewise for *v*). $R_{j}^{1}$ is as *R_j _*but the last character must be from the string *B*^1^. $R_{j}^{2}$ is defined similarly.

**Lemma 1**. Let *A, B*^1 ^and *B*^2 ^be strings and *R_j_*, $R_{j}^{1}$ and $R_{j}^{2}$ as above. Now

$$\text{ed}\left(A_{i},\;R_{j}\right)=\text{min}\left\{\begin{array}{c} \text{ed}\left(A_{i},\;R_{j}^{1}\right)\\ \text{ed}\left(A_{i},\;R_{j}^{2}\right). \end{array}\right)$$

**Algorithm 1 **Haploid to diploid alignment algorithm.

**  function **DIPLOID _ALIGN(*A, B*^1^*, B*^2^*, m*^1^*, m*^2^*, reference_length*)

   *d*^1^[length(*B*^1^) + 1][length(*A*) + 1]

   *d*^2^[length(*B*^2^) + 1][length(*A*) + 1]

    /* Initialise as in Levenshtein distance. */

  **function **CALCULATE_COLUMN(*matrix, col, B_char*)

    **for ***i ← *1, length(*A*) **do**

     

$$matrix\left[col\right]\left[i\right]\leftarrow \text{min}\left\{\begin{array}{c} matrix\left[col-1\left[i\right]+1\right]\\ matrix[col-1\left[i-1\right]+\left[A\left[i\right]\neq B\text{\_}char\right]\\ matrix\left[col\right]\left[i-1\right]+1 \end{array}\right)$$

  **function **MIN_FROM_TO(*from, to*)

    **for ***i *= 1 *→ *length(*A*) **do**

     

$$to\left[i\right]\leftarrow \text{min}\left\{\begin{array}{c} to\left[i\right]\\ from\left[i\right] \end{array}\right)$$

  *column1 ← *1

  *column2 ← *1

  **for ***j ← *0*, reference_length − *1 **do**

    **if ***m*^1^[*column1 − *1] = *j ***then**

CALCULATE_COLUMN(*d*^1^*, column1, B*^1^[*column1 − *1])

     *column1 ← column1 *+ 1

    **if ***m*^2^[*column2 − *1] = *j ***then**

CALCULATE_COLUMN(*d*^2^*, column2, B*^2^[*column2 − *1])

      *column2 ← column2 *+ 1

    **if ***m*^1^[*column1 − *2] = *j *and (*m*^2^[*column2 − *1] = *j *+ 1 or *m*^2^[*column2 − *2] = *j*) **then**

MIN_FROM_TO(*d*^1^[*column1 − *1]*, d*^2^[*column2 − *1])

    **if ***m*^2^[*column2 − *2] = *j *and (*m*^1^[*column1 − *1] = *j *+ 1 or *m*^1^[*column1 − *2] = *j*) **then**

MIN_FROM_TO(*d*^2^[*column2 − *1]*, d*^1^[*column1 − *1])

    **while ***m*^1^[*column1 − *1] *≤ j ***do**

CALCULATE_COLUMN(*d*^1^*, column1, B*^1^[*column1 − *1])

      *column1 ← column1 *+ 1

    **while ***m*^2^[*column2 − *1] *≤ j ***do**

CALCULATE_COLUMN(*d*^2^*, column2, B*^2^[*column2 − *1])

      *column2 ← column2 *+ 1

 

$$return\text{min}\left\{\begin{array}{c} d^{1}\left[\text{length}\left(B^{1}\right)\right]\left[\text{length}\left(A\right)\right]\\ d^{2}\left[\text{length}\left(B^{2}\right)\right]\left[\text{length}\left(A\right)\right] \end{array}\right)$$

**Lemma 2**. When a recombination

$$B^{2}\left[0\ldots \upsilon -2\right]B^{1}\left[k-1\ldots \right]$$

is possible, that is,

$$m^{2}\left[\upsilon -2\right]+1=m^{1}\left[k-1\right]\;\text{or}\;m^{2}\left[\upsilon -2\right]=m^{1}\left[k-2\right],$$

it holds that

$$\begin{array}{c} \text{min}\left\{\begin{array}{c} \text{min}\left\{\begin{array}{c} \text{ed}\left(A_{i},R_{m^{1}\left[k-2\right]}^{1}\right)\\ \text{ed}\left(A_{i},R_{m^{2}\left[\upsilon -2\right]}^{2}\right) \end{array}\right)+1\\ \text{min}\left\{\begin{array}{c} \text{ed}\left(A_{i-1},R_{m^{1}\left[k-2\right]}^{1}\right)\\ \text{ed}\left(A_{i-1},R_{m^{2}\left[\upsilon -2\right]}^{2}\right) \end{array}\right)+\delta \\ \text{ed}\left(A_{i-1},\;R_{m^{1}\left[k-1\right]}^{1}\right)+1 \end{array}\right)\\ =\text{ed}\left(A_{i},R_{m^{1}\left[k-1\right]}^{1}\right) \end{array}$$

where *δ *= 1 if *A*[*i*] *≠ B*^1^[*k − *1] and 0 otherwise.

*Proof*. First we observe that

$$m^{\text{2}}\left[\upsilon \;-\text{2}\right]\;+\text{ 1 }=m^{\text{1}}\left[k\;-\text{1}\right]$$

implies

$$m^{1}\left[k\;-\text{2}\right]\leq m^{\text{2}}\left[\upsilon \;-\text{2}\right]\;=m^{\text{1}}\left[k\;-\text{1}\right]-\text{1}.$$

Using this we get

$$\begin{array}{c} \text{min}\left\{\begin{array}{c} \text{min}\left\{\begin{array}{c} \text{ed}\left(A_{i},R_{m^{1}\left[k-2\right]}^{1}\right)\\ \text{ed}\left(A_{i},R_{m^{2}\left[\upsilon -2\right]}^{2}\right) \end{array}\right)+1\\ \text{min}\left\{\begin{array}{c} \text{ed}\left(A_{i-1},R_{m^{1}\left[k-2\right]}^{1}\right)\\ \text{ed}\left(A_{i-1},R_{m^{2}\left[\upsilon -2\right]}^{2}\right) \end{array}\right)+\delta \\ \text{ed}\left(A_{i-1},\;R_{m^{1}\left[k-1\right]}^{1}\right)+1 \end{array}\right)\\ =\text{min}\left\{\begin{array}{c} \text{min}\left\{\begin{array}{c} \text{ed}\left(A_{i},R_{m^{2}\left[\upsilon -2\right]}^{1}\right)\\ \text{ed}\left(A_{i},R_{m^{2}\left[\upsilon -2\right]}^{2}\right) \end{array}\right)+1\\ \text{min}\left\{\begin{array}{c} \text{ed}\left(A_{i-1},R_{m^{2}\left[\upsilon -2\right]}^{1}\right)\\ \text{ed}\left(A_{i-1},R_{m^{2}\left[\upsilon -2\right]}^{2}\right) \end{array}\right)+\delta \\ \text{ed}\left(A_{i-1},\;R_{m^{1}\left[k-1\right]}^{1}\right)+1 \end{array}\right) \end{array}$$

and by Lemma 1

$$\begin{array}{c} =\text{min}\left\{\begin{array}{c} ed\;\left(A_{i},\;{R_{m^{2}}}_{\left[\upsilon -2\right]}\right)+1\\ ed\left(A_{i-1},\;R_{m^{2}\left[\upsilon -2\right]}\right)+\delta \\ ed\left(A_{i-1},\;{R^{1}}_{m^{1}\left[k-1\right]}\right)+1 \end{array}\right)\\ =ed\left(A_{i},{R^{1}}_{m^{1}\left[k-1\right]}\right) \end{array}$$

which is as claimed. Note that if *m*^2^[*v − *2] = *m*^1^[*k − *2], the claim follows directly without the observation.

**Lemma 3**. If the strings *B*^1 ^and *B*^2 ^do not overlap, that is

$$\begin{array}{c} m^{\text{1}}\left[\text{length}\left(B^{\text{1}}\right)-\text{1}\right]<\;m^{\text{2}}\left[0\right]\text{ or}\\ m^{}\left[\text{length}\left(B^{\text{2}}\right)-\text{1}\right]<\;m^{\text{1}}\left[0\right] \end{array}$$

then we can compute the diploid_align as the Levenshtein distance between the string *A *and the appropriate concatenation *B*^1^*B*^2 ^or *B*^2^*B*^1^.

**Theorem 1**. The Algorithm 1 calculates

$$\begin{array}{c} d^{1}\left[k\right]\left[i\right]=\text{ed}(A_{i},R_{m^{1}\left[k-1]\right)}^{{}_{1}}1)\text{ and}\\ d^{2}\left[v\right]\left[i\right]=\text{ed}\left(A_{i},R_{m^{2}\left[\upsilon -1,]\right)}^{2}\right). \end{array}$$

*Proof*. The proof is by induction over the index sum *i *+ *k *for the case

$$d^{\text{1}}\left[k\right]\left[i\right]\;=\text{ ed}\left(A_{i},{R^{\text{1}}}_{m^{\text{1}}\left[k-\text{1]}\right)}\right).$$

The other case is symmetric.

First consider the base cases. The algorithm initialises the matrices as in the Levenshtein distance algorithm. By Lemma 3 we can assume that the strings *B*^1 ^and *B*^2 ^overlap and thus no recombination needs to be considered before the first iteration of the algorithm. The base cases are thus as required. Let us assume that the claim holds for *i *+ *k < p *+ *q*. If the condition

(1) $$\begin{array}{c} m^{\text{2}}\left[column2\;-\text{2}\right]\;=\;=j\;\text{and}\\ (m^{1}\left[column1-1\right]=\;=j+1\;\text{or}\\ m^{1}\left[column1-1\right]=\;=j) \end{array}$$

held on a previous iteration, then the minimum of the columns *d*^1^[*q − *1] and *d*^2^[*column2 − *1] was assigned to *d*^1^[*q − *1]. It also means that

(2) $$m^{\text{2}}\left[column2\;-\text{2}\right]\;+\text{ 1 }=m^{\text{1}}\left[q\;-\text{1}\right].$$

Let *δ *= 1 if *A*[*p*] ≠ *B*^1^[*q − *1] and 0 otherwise. Now the algorithm calculates

$$\begin{array}{c} d^{1}\left[q\right]\left[p\right]=\text{min}\left\{\begin{array}{c} \text{min}\left\{\begin{array}{c} d^{1}\left[q-1\right]\;\left[p\right]\\ d^{2}\left[column2-1\right]\;\left[p\right] \end{array}\right)+1\\ \text{min}\left\{\begin{array}{c} d^{1}\left[q-1\right]\;\left[p-1\right]\\ d^{2}\left[column2-1\right]\;\left[p-1\right] \end{array}\right)+\delta \\ d^{1}\left[q\right]\;\left[p-1\right]+1 \end{array}\right)\\ \;=\text{min}\left\{\begin{array}{c} \text{min}\left\{\begin{array}{c} \text{ed}\left(A_{p},R_{m^{1}\left[q-2\right]}^{1}\right)\\ \text{ed}\left(A_{p},R_{m^{2}\left[column2-2\right]}^{2}\right) \end{array}\right)+1\\ \text{min}\left\{\begin{array}{c} \text{ed}\left(A_{p-1},R_{m^{1}\left[q-2\right]}^{1}\right)\\ \text{ed}\left(A_{p-1},R_{m^{2}\left[column2-2\right]}^{2}\right) \end{array}\right)+\delta \\ \text{ed}\left(A_{p-1},\;R_{m^{1}\left[q-1\right]}^{1}\right)+1 \end{array}\right)\\ \;=\text{ed}\left(A_{p},R_{m^{1}\left[q-1\right]}^{1}\right) \end{array}$$

where the first equality holds by the induction assumption. The second equality holds as by equation (2) we can use Lemma 2 with the assignment *v *:= *column2 *and *k *:= *q*.

If condition (1) did not hold on the previous iteration, no minimisation was performed. Thus the algorithm calculates

$$\begin{array}{c} d^{1}\left[q\right]\;\left[p\right]=\text{min}\left\{\begin{array}{c} d^{1}\;\left[q-1\right]\;\left[p\right]+1\\ d^{1}\left[q-1\right]\;\left[p-1\right]+\delta \\ d^{1}\left[q\right]\;\left[p-1\right]+1 \end{array}\right)\\ \;=\text{min}\left\{\begin{array}{c} \text{ed}\;\left(A_{p},\;{R^{1}{}_{m^{1}}}_{\left[q-2\right]}\right)+1\\ \text{ed}\left(A_{p-1},\;{R^{1}}_{m^{2}\left[q-2\right]}\right)+\delta \\ \text{ed}\left(A_{p-1},\;{R^{1}}_{m^{1}\left[q-1\right]}\right)+1 \end{array}\right)\\ \;=\text{ed}\left(A_{p},{R^{1}}_{m^{1}\left[q-1\right]}\right) \end{array}$$

which is correct, as no recombination could be made at this point.

#### Diagonal doubling

The runtime optimisation known as *diagonal doubling *[6] can be used also with Algorithm 1. The idea of the original algorithm is as follows. Consider checking whether the Levenshtein edit distance between two sequences of length *n *is at most *k*, where *k *is a given cutoff. Then it is enough to do computation on diagonals *i − j *such that *|i − j| ≤ *[*k*/2], since every change of diagonal costs 1; any path visiting a cell outside this diagonal zone results into edit script with cost more than *k*. With unknown cutoff, it is easy to see that starting with *k *= 1, doubling the value of *k *at each iteration, recomputing the diagonal zone, and stopping the doubling when the computed distance value remains unchanged, results in an *O *(*dn*) time algorithm, where *d *is the edit distance. The idea works with technical changes for two sequences of different length. With some care, one can perform the computation in *O *(*d*) space.

To modify the optimisation for our haploid to diploid alignment, the following details need to be taken into account. Because the two columns calculated by the algorithm at every iteration may not completely overlap, one needs to make sure that the minimisation step is done correctly. The minimum of the two columns can only be taken for the overlapping part. Because of the two Levenshtein calculations, we also need to choose the starting cutoff values with care. In particular one needs to start with *max*(*|*length(*A*) *− *length(*B*^1^)*|, |*length(*A*) *− *length(*B*^2^)*|*), so that the diagonal is big enough to cover the bottom right corner in both matrices.

The diagonal doubling does not affect the correctness of the Algorithm 1. The same argument as in the case of Levenshtein distance applies here. Let us assume that we are interested only on edit distances less than a certain cutoff value. As both matrices contain a Levenshtein distance, it must be that the values grow monotonically when we deviate from the diagonal. Thus, when taking the minimum between the two columns, the values outside the overlapping area must be greater than the current cutoff value. This means that the optimum edit distance must derive from the values in the overlapping area.

## Results and discussion

To test the algorithm, we implemented it in C with the diagonal doubling optimisation [6]. The diploid_align algorithm was first tested with generated data. Two diploid strings were generated from a DNA string of appropriate length. Single inserts, deletes and substitutions were applied each with probability of 0.003, and with probability 0.5 they were applied to both diploids. This gives approximately *n/*100 variations in a string of length *n*. The algorithm was then run five times with the original string as the haploid input and an average of the runtimes was taken. The test machine was a laptop with a Intel Core 2 Duo T7300 2GHz processor running Linux. The results are shown in the Table 1. The actual distance is always about half the generated amount of variations, as nearly half the variations are heterozygous and in those cases one allele has the same base as the haploid input (original sequence).

**Table 1:** Results of running diploid_align with generated data.

input size	runtime in seconds	variations	distance
10000	0.014000	95	48
20000	0.054000	190	90
30000	0.168000	291	145
40000	0.258000	382	190
50000	0.446000	478	236
60000	0.562000	561	281
70000	0.712000	656	329
80000	1.122000	745	377
90000	1.262000	843	419
100000	1.748000	931	460
1000000	148.619995	10051	4983

The second test used artificially generated diploids from human chromosome 20 created for a pilot variation calling challenge, Variathon 2013 [3]. From each of the two submissions taking part in the challenge we created one predicted haploid by a simple script that applied the predicted variants to the reference genome. These predicted haploids were then aligned to the diploid. Table 2 shows the original evaluations from the challenge (first 6 columns) and our new evaluation with edit distance. As can be seen, our evaluation agrees with the evaluation done for the challenge.

**Table 2:** Results with predicted haploids from Variathon.

haploid	tp	fp	fn	precision	recall	edit distance	runtime in minutes
GBT	66190	323	29714	0.995	0.690	23232	690
BoBiocomp	86589	2288	9267	0.974	0.903	17422	380

We also ran a small experiment to highlight the issue of invariance. With the example given in Section Methods, Invariance, the evaluation method used in Variathon 2013 [3] finds only two common variations, as claimed. Not surprisingly, the edit distance between the diploid and the haploid is zero, as in both cases the variations when combined produce the same genome **GTCCCATAAG**.

## Conclusions

We proposed an approach for assessing variation calling predictions in the case of artificial diploid genomes, using a modification of global alignment with a diagonal doubling optimisation to compute it on large inputs. The motivation for the approach was to avoid the invariance problems of direct variation comparison.

We tested the approach on a haploid instance to show its robustness and scalability to complete (small) genomes. We also compared the output of our algorithm with applicable evaluations from a variation calling challenge and the results agreed.

## Competing interests

The authors declare that they have no competing interests.

## Authors' contributions

VM developed the idea. JR worked out the details of the algorithm, implemented it, and ran the experiments. Both authors contributed to the writing. Both authors read and approved the final manuscript.

## Declarations

Publication of this article was supported by the Academy of Finland under grant 250345 (CoECGR).

This article has been published as part of BMC Bioinformatics Volume 14 Supplement 15, 2013: Proceedings from the Eleventh Annual Research in Computational Molecular Biology (RECOMB) Satellite Workshop on Comparative Genomics. The full contents of the supplement are available online at http://www.biomedcentral.com/bmcbioinformatics/supplements/14/S15.
